# Myristic Acid Supplementation Aggravates High Fat Diet-Induced Adipose Inflammation and Systemic Insulin Resistance in Mice

**DOI:** 10.3390/biom12060739

**Published:** 2022-05-24

**Authors:** Viswanathan Saraswathi, Narendra Kumar, Weilun Ai, Thiyagarajan Gopal, Saumya Bhatt, Edward N. Harris, Geoffrey A. Talmon, Cyrus V. Desouza

**Affiliations:** 1 Division of Diabetes, Endocrinology, and Metabolism, Department of Internal Medicine, University of Nebraska Medical Center, Omaha, NE 68198, USA; narendra30@gmail.com (N.K.); weilun.ai@unmc.edu (W.A.); mailthiyagarajang@gmail.com (T.G.); saumya.bhatt@unmc.edu (S.B.); cdesouza@unmc.edu (C.V.D.); 2 Research Service, VA Nebraska-Western Iowa Health Care System, Omaha, NE 68105, USA; 3 Department of Biochemistry, University of Nebraska, Lincoln, NE 68588, USA; eharris5@unl.edu; 4 Department of Pathology and Microbiology, University of Nebraska Medical Center, Omaha, NE 68198, USA; gtalmon@unmc.edu

**Keywords:** myristic acid, saturated fatty acids, obesity, insulin resistance, adipose inflammation

## Abstract

Saturated fatty acids (SFAs) are considered to be detrimental to human health. One of the SFAs, myristic acid (MA), is known to exert a hypercholesterolemic effect in mice as well as humans. However, its effects on altering adipose tissue (AT) inflammation and systemic insulin resistance (IR) in obesity are still unclear. Here, we sought to determine the effects of a high fat (HF) diet supplemented with MA on obesity-associated metabolic disorders in mice. Wild-type C57BL/6 mice were fed a HF diet in the presence or absence of 3% MA for 12 weeks. Plasma lipids, plasma adipokines, AT inflammation, systemic IR, glucose homeostasis, and hepatic steatosis were assessed. The body weight and visceral adipose tissue (VAT) mass were significantly higher in mice receiving the HF+MA diet compared to HF diet-fed controls. Plasma total cholesterol levels were marginally increased in HF+MA-fed mice compared to controls. Fasting blood glucose was comparable between HF and HF+MA-fed mice. Interestingly, the plasma insulin and HOMA-IR index, a measure of insulin resistance, were significantly higher in HF+MA-fed mice compared to HF controls. Macrophage and inflammatory markers were significantly elevated in the AT and AT-derived stromal vascular cells upon MA feeding. Moreover, the level of circulating resistin, an adipokine promoting insulin resistance, was significantly higher in HF+MA-fed mice compared with HF controls. The insulin tolerance test revealed that the IR was higher in mice receiving the MA supplementation compared to HF controls. Moreover, the glucose tolerance test showed impairment in systemic glucose homeostasis in MA-fed mice. Analyses of liver samples showed a trend towards an increase in liver TG upon MA feeding. However, markers of oxidative stress and inflammation were reduced in the liver of mice fed an MA diet compared to controls. Taken together, our data suggest that chronic administration of MA in diet exacerbates obesity-associated insulin resistance and this effect is mediated in part, via increased AT inflammation and increased secretion of resistin.

## 1. Introduction

Consumption of saturated fatty acids (SFAs) is strongly associated with metabolic syndrome and cardiovascular disease (CVD) (reviewed in [1,2,3]). However, SFAs appear to exert differential effects on metabolic disorders, depending on their chain length. For example, the short- and medium-chain SFAs are beneficial against metabolic disorders [4,5] whereas, the long-chain SFAs are considered to increase the risk for CVD [6]. Regarding the long-chain SFAs, the effects of palmitic acid (a 16-carbon fatty acid) on metabolic diseases have been well-studied [1,7]. However, the role of MA, a 14-carbon fatty acid, in altering obesity-related metabolic disorders remains unclear.

Palmitic acid (PA), a 16-carbon fatty acid, is the most common SFA, accounting for 20–30% of total fatty acids in the human body [8]. On the other hand, myristic acid (MA), a 14-carbon fatty acid, is present at relatively low concentrations in the tissues of humans and animals. On average, MA accounts for only 1% of all endogenous fatty acids. Although it is a minor fatty acid, it has attracted growing attention because of clinical evidence for its association with metabolic disorders and CVD. In fact, MA has been identified in serum and/or liver after lipidomics analysis in mouse models and humans with non-alcoholic fatty liver disease (NAFLD) and non-alcoholic steatohepatitis (NASH) [9,10,11]. Moreover, MA is involved in the myristoylation of proteins, a post-translational modification, altering various signaling pathways, thereby altering cellular functions [12,13].

Regarding the link between MA and metabolic disorders, previous observational studies showed that the MA content in serum triglycerides was higher in metabolically unhealthy obese individuals compared to lean healthy and metabolically healthy obese subjects [14]. Serum levels of MA were positively correlated to obesity and obesity-associated systolic blood pressure [15,16]. Diet and/or exercise reduced plasma MA levels in subjects with metabolic syndrome [17,18]. A strong link exists between MA and CVD as well. For example, human interventional studies showed that a diet rich in MA resulted in hypercholesterolemia [19,20,21]. A number of animal studies have also shown that MA is hypercholesterolemic [22,23]. However, some studies suggest that MA can be beneficial against metabolic disorders. For example, MA increased high-density lipoprotein (HDL) cholesterol without raising the low-density lipoprotein (LDL) cholesterol level in human subjects [24]. Moreover, in a study using a congenital model of type 2 diabetes, MA attenuated hyperglycemia [25]. Thus, the effect of MA on a systemic metabolic profile is still unclear. Moreover, while a number of studies addressed the effect of MA on liver lipid metabolism, its role in altering obesity-linked insulin resistance (IR) remains unclear. Therefore, using a model of diet-induced obesity, we sought to determine the role of purified MA supplementation in altering IR and systemic metabolic homeostasis in mice.

## 2. Materials and Methods

*Mice and diet*. Eight ten wk old, male wild-type C57BL/6 mice were purchased from Jackson Lab. The high fat (HF) diet containing 45% fat calories and the high fat diet supplemented with 3% MA were obtained from Research Diets Inc. For MA supplementation, a custom diet was prepared by replacing 3% lard in the HF diet with 3% (*w*/*w*) purified MA (Nu-Chek Prep, Elysian, MN, USA). The diet composition is shown in Table 1. Based on the fatty acid composition of individual fat in the diet, the HF diet had 0.23% MA (from lard and soybean oil) and the HF+MA diet had 3.2% MA (from MA, lard, and soybean oil). Mice were fed a HF diet or HF+MA diet for 12 weeks. All animal procedures were carried out with approval from the Institutional Animal Care and Use Committee of VA Nebraska-Western Iowa Health Care System.

*Plasma metabolic variables*. Fasting blood glucose was determined using the Accu-Chek Aviva glucometer (Roche Diabetes Care, Inc., IN, USA). Plasma total cholesterol and free fatty acids (FFA) were analyzed using kits from (Cliniqa, San Marcos, CA) and Wako (FUJIFILM Wako Chemicals, Richmond, VA, USA), respectively. Plasma insulin was measured using a kit from Mercodia (Winston Salem, NC, USA). The homeostasis model of assessment of insulin resistance (HOMA-IR) was calculated using the formula: fasting glucose (milligrams per deciliter) × fasting insulin (microunits per milliliter)/405 [26].

*Plasma adipokines*. Circulating levels of leptin, adiponectin, and resistin were determined in plasma by Luminex xMAP Technology using a Mouse Metabolic Magnetic Bead Panel (#MADKMAG-71K, MilliporeSigma, Burlington, MA, USA) as we reported earlier [27]. Briefly, 10 µL of plasma samples were mixed with sonicated beads for each marker along with a matrix solution and assay buffer in a 96-well plate and incubated overnight on an orbital shaker (110 rpm) at 4 °C. The following day, detection antibodies were added and a magnetic plate holder was used to retain the beads during the washing steps. The quantification of beads with respective standards and samples was measured by a Luminex MAGPIX software, xPONENT 4.2.1324.0 (version 1, Burlington, MA, USA).

*Intra-peritoneal insulin tolerance test*. To determine the ability of mice in responding to insulin, we performed the insulin tolerance test (ITT) as we reported earlier [28,29]. Briefly, 10 wk post-diet, mice were fasted for 5 h and injected with an acute bolus of insulin intra-peritoneally at 0.5 U/kg body weight. Blood glucose levels were monitored at various time points up to 2 h.

*Intra-peritoneal glucose tolerance test*. For the glucose tolerance test (GTT), 11 wk post-diet, mice were fasted for 5 h and injected with an acute bolus of glucose at 1 g/kg body weight. Blood glucose levels were monitored at various time points up to 2 h. GTT was performed using the method we reported previously [28,29].

*Liver lipid profile*. Liver triglycerides (TG) and cholesterol esters were determined by gas chromatographic analysis at the Lipid Core Laboratory of Vanderbilt University as we reported earlier [30].

*Isolation of stromal vascular cells (SVCs)*. About 500 mg of visceral adipose tissue (VAT) samples were minced and incubated with collagenase Type II (Sigma-Aldrich, St. Louis, MO, USA) at 1 mg/mL for 1 h. The cell suspension was filtered through a 100 µm nylon filter. After centrifugation, the SVCs in the pellet were collected.

*Real-time polymerase chain reaction*. The mRNA levels of inflammatory genes and those regulating lipid metabolism were analyzed by a two-step real-time PCR analysis. Briefly, the total RNA was extracted from liver, VAT, and SVCs using the Trizol RNA extraction kit. About 400 ng of RNA was reverse transcribed to cDNA using the iScript Supermix from Bio-Rad (Hercules, CA, USA). Primer probes for various genes encoding for macrophage and inflammatory markers, M2 markers, adipokines, and lipid metabolism were purchased from Applied Biosystems (Table 2). The mRNA levels of these genes were determined and normalized to 18S. A ΔΔCT method was used to determine the levels of individual genes.

*Histology*. Liver sections were fixed in 10% formalin and paraffin blocks were prepared. The liver was cut into 4 µm sections, which were stained with hematoxylin and eosin (H&E) to determine liver morphology. Liver sections were viewed and scored for steatosis, immune cell infiltration, and ballooning by Dr. Geoffrey Talmon, a board-certified pathologist.

For immunofluorescence, after an overnight incubation with anti-F4/80 primary antibody (Cell Signaling Tech) at 4 °C, the VAT sections (10 µm) were washed and incubated with FITC-conjugated secondary antibody for 1 h at room temperature. Autofluorescence was quenched using 0.1% Sudan Black B in 70% ethanol. The sections were mounted with ProLong (Waltham, MA, USA) gold antifade 4′,6-diamidino-2-phenylindole (DAPI).

MCP1 and MIP1α in VAT sections were detected by immunohistochemistry. Briefly, deparaffinized tissue sections were incubated overnight with anti-MCP1 or anti-MIP1α primary antibody (Thermofisher Scientific, Waltham, MA, USA) at 4 °C. Sections were then incubated for 1 hr with biotinylated secondary antibody (Vector IHC kit, Newark, CA, USA) at room temperature. Vectastain ABC reagent (Avidin-Biotinylated HRP) was added for 30 min followed by incubation with peroxidase substrate (3,3′-diaminobenzidine (DAB), Vector kit) until color developed. Sections were counterstained with hematoxylin. For both immunofluorescence and immunohistochemistry analyses, images (20×) were captured using the Nikon Eclipse 80i inverted microscope (Melville, NY, USA.

*Hepatic oxidative stress*. Lipid peroxidation was assessed by measuring thiobarbituric acid-reactive substances (TBARS). We also measured hepatic glutathione (GSH) content. TBARS and GSH were determined using kits from Cayman Chemicals.

*Statistics*. Statistical significance was determined by a two-tailed Student’s *t*-test. For differences in blood glucose levels during ITT and GTT, a two-way analysis of variance (time and treatment) was performed and the significance was determined using Bonferroni’s *post-hoc* test. Graph-Pad Prism software version 8.0 (San Diego, CA, USA) was used to determine statistical significance (*p* < 0.05 was considered significant).

## 3. Results

**Figure 1:**
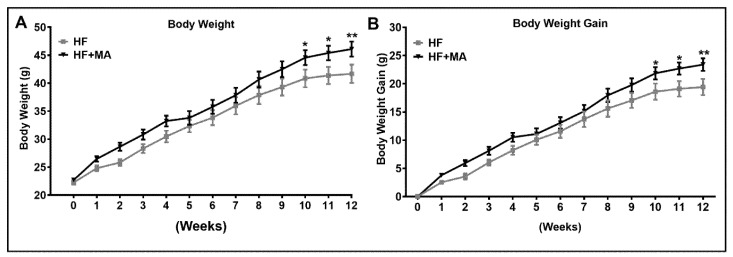
**Effect of dietary myristic acid on body weight.** Wild-type C57BL/6 mice were fed a HF diet supplemented with MA for 12 weeks. Weekly body weight (**A**) and body weight change from baseline (**B**) were recorded during the feeding period. Values are expressed as mean ± SEM of 8 mice per group. * *p* < 0.05 and ** *p* < 0.01 vs. HF. HF, high fat; HF+MA, high fat + myristic acid.

*Effect of dietary myristic acid on body weight*. We determined the body weight and body weight change from baseline in HF- and HF+MA-fed mice during the feeding period. Our data clearly show that mice fed a HF+MA diet showed a significant increase in total body weight as well as body weight gain at 10, 11, and 12 weeks compared to HF diet-fed control mice (Figure 1A,B). However, the daily food intake did not alter between the two groups (2.9 and 3.1 g/mouse/day in HF and HF+MA groups, respectively), indicating that the increase in body weight is due to a change in energy metabolism by MA and not by increasing energy intake.

**Figure 2:**
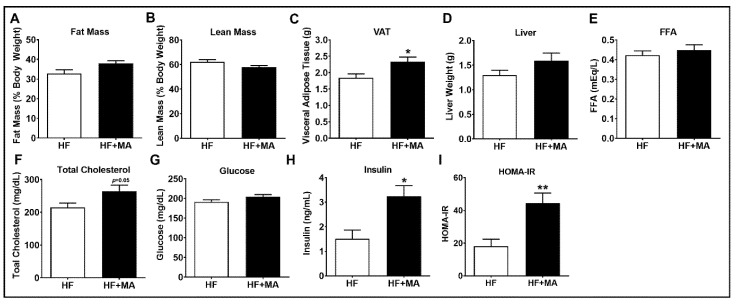
**Effect of dietary myristic acid on metabolic variables.** Total fat mass (**A**) and lean mass (**B**) were assessed in live mice using the EchoMRI body composition analyzer. VAT (**C**) and liver weights (**D**) were determined at sacrifice. Plasma-free fatty acids (**E**) and total cholesterol (**F**) were measured. Fasting blood glucose (**G**), plasma insulin (**H**), and HOMA-IR (**I**) were assessed. Values are expressed as mean ± SEM of 7–8 samples per group. * *p* < 0.05 and ** *p* < 0.01 vs. HF. HF, high fat; HF+MA, high fat + myristic acid; VAT, visceral adipose tissue; FFA, free fatty acid.

*Effect of dietary myristic acid on metabolic variables*. We next analyzed the fat mass using the EchoMRI body composition analyzer. We noted that the total fat mass and lean mass did not alter significantly between groups (Figure 2A,B). The VAT mass was significantly higher in the HF+MA group compared to HF-fed controls (Figure 2C). The liver weight and plasma FFAs did not change significantly between groups (Figure 2D,E). A marginal increase in plasma total cholesterol was noted (*p* = 0.05) in HF+MA-fed mice compared to HF-fed controls (Figure 2F). The fasting blood glucose was comparable between groups (Figure 2G). Interestingly, the plasma insulin level was significantly higher in HF+MA-fed mice compared to controls (Figure 2H). Moreover, the HOMA-IR index was significantly higher in the HF+MA group compared to HF-fed mice (Figure 2I).

**Figure 3:**
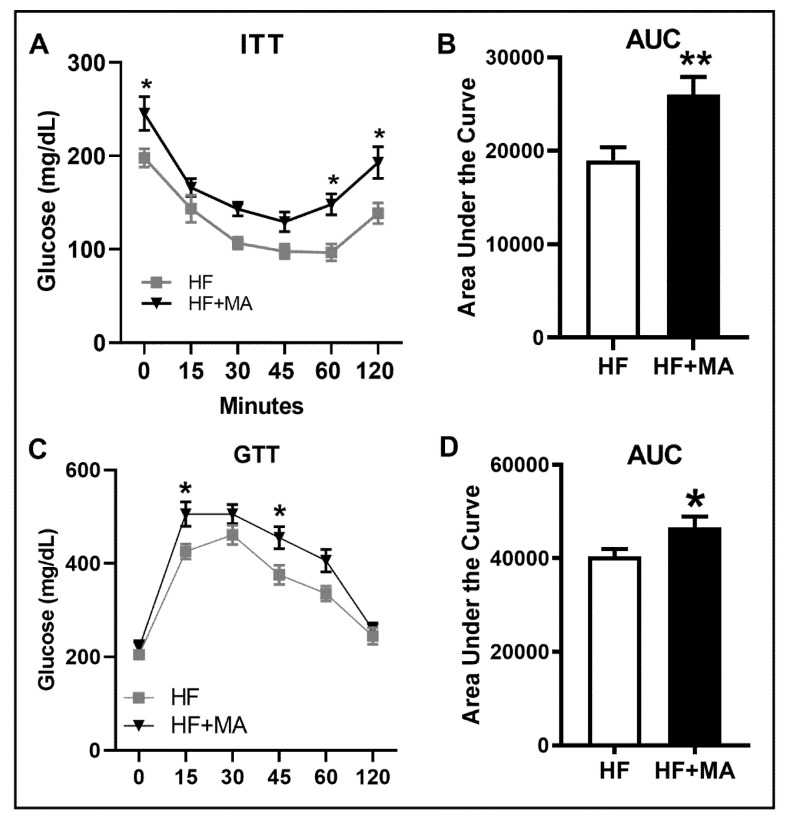
**Effect of dietary myristic acid on systemic insulin resistance and glucose homeostasis.** Intra-peritoneal insulin tolerance test was performed to assess systemic insulin resistance. Ten wk post-diet, mice were injected (IP) with insulin at 0.5 U/kg body weight. Blood glucose levels were measured at indicated time points (**A**). The area under the curve was measured and plotted (**B**). Intra-peritoneal glucose tolerance test was conducted to determine systemic glucose homeostasis. Eleven wk post-diet, mice were injected (IP) with glucose at 1 g/kg body weight. Blood glucose levels were measured at indicated time points (**C**). The area under the curve was measured and plotted (**D**). Values are expressed as mean ± SEM of 8 samples per group. * *p* < 0.05 and ** *p* < 0.01 vs. HF. HF, high fat; HF+MA, high fat + myristic acid; ITT, insulin tolerance test; GTT, glucose tolerance test; AUC, area under the curve.

*Effect of dietary myristic acid on systemic IR*. We performed an ITT to assess the ability of mice in responding to insulin. Both groups showed a decline in blood glucose levels after insulin administration. However, the blood glucose levels were higher in mice that received the HF+MA diet compared to mice fed a HF control diet (Figure 3A). The area under the curve values was also significantly higher in mice that received the MA supplementation compared to control mice (Figure 3B). In order to determine how the mice respond to an acute bolus of glucose, we performed a GTT. We noted that the blood glucose levels were higher in mice that received the HF+MA diet compared to HF controls (Figure 3C). Moreover, the area under the curve values was significantly higher in HF+MA-fed mice compared to controls (Figure 3D). Taken together, these data suggest that MA promotes systemic IR and glucose intolerance in obesity.

**Figure 4:**
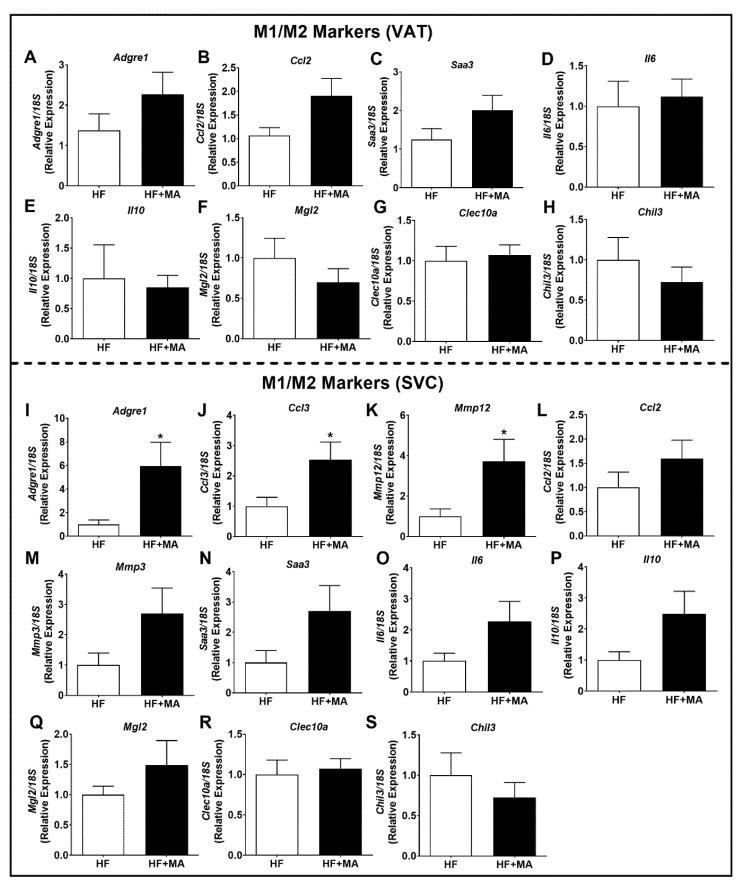
**Effect of dietary myristic acid on M1 and M2 markers in VAT and VAT-derived stromal vascular cells.** Real-time PCR analysis was performed in the VAT for the mRNA levels of *Adgre*, a macrophage marker (**A**), other inflammatory genes (**B**–**D**), and M2 markers (**E**–**H**). Stromal vascular cells from the VAT were isolated and real-time PCR analysis was performed for macrophage (**I**) and inflammatory markers (**J**–**O**) as well as M2 markers (**P**–**S**). Values are expressed as mean ± SEM of 7–8 samples in each group. * *p* < 0.05 vs. HF. VAT, visceral adipose tissue; HF, high fat; HF+MA, high fat + myristic acid; SVC, stromal vascular cells.

*Effect of dietary myristic acid on M1 and M2 markers in AT*. We first performed a real-time PCR analysis in VAT samples to determine the mRNA levels of macrophage and inflammatory markers, indicating the M1 macrophage polarization. The mRNA levels of macrophage (*Adgre*, encoding F4/80) and inflammatory markers did not alter significantly between groups (Figure 4A–D). Moreover, various markers of M2 macrophage polarization, including *Il10*, *Mgl2*, *Clec10a*, and *Chil3*, did not alter significantly between groups in VAT (Figure 4E–H). On the other hand, the real-time PCR analysis of the SVCs collected from the VAT showed that the mRNA levels of *Adgre* (a macrophage marker) were significantly higher (*p* < 0.05) in HF+MA-fed mice compared to HF controls (Figure 4I). In addition, the mRNA levels of inflammatory markers, including *Ccl3* (encoding MIP1α) and *Mmp12*, were significantly elevated (*p* < 0.05) (Figure 4J–K) and those of *Ccl2*, *Mmp3*, *Saa3*, and *Il6* did not alter significantly in the HF+MA group (Figure 4L–O). However, the M2 markers did not differ between the HF and HF+MA groups (Figure 4P–S).

**Figure 5:**
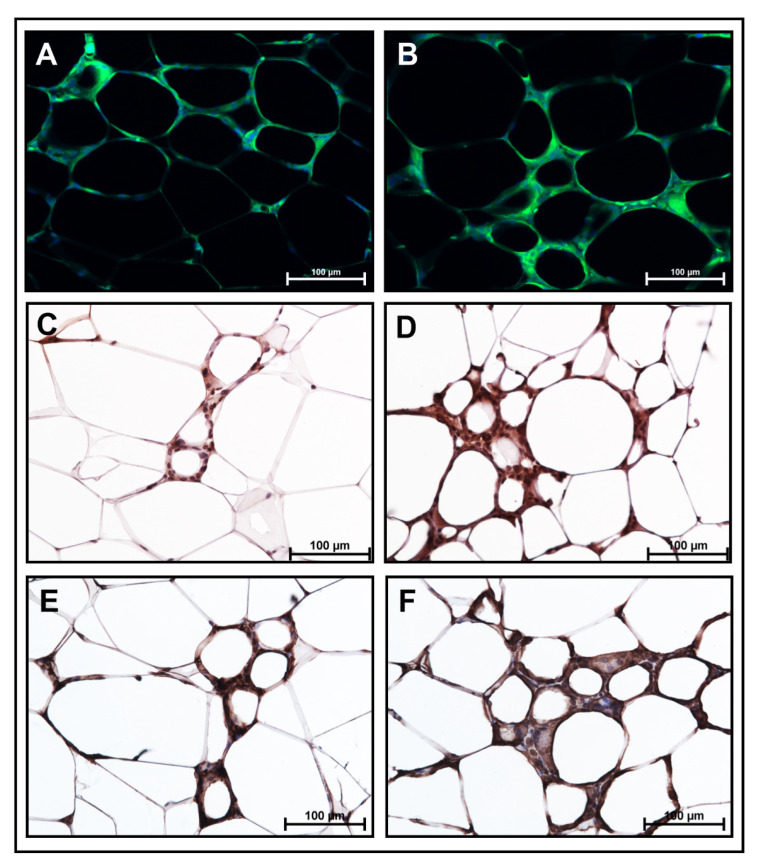
**Immunostaining of VAT sections from HF and HF+MA-fed mice for macrophage and inflammatory markers.** VAT sections from HF and HF+MA-fed mice were stained for F4/80 (**A**,**B**), MCP1 (**C**,**D**), and MIP1α (**E**,**F**). Images were captured at 20× magnification using the Nikon Eclipse 80i microscope. HF, high fat; HF+MA, high fat + myristic acid.

We further validated these data by performing immunostaining in VAT sections. Our data clearly show that F4/80, a macrophage marker, showed a marked increase in HF+MA-fed mice compared to HF controls (Figure 5A,B). Moreover, MCP1 (Figure 5C,D) and MIP1α (Figure 5E,F), the inflammatory markers, were higher in the HF+MA group compared to HF-fed mice. These data suggest that adding MA to the HF diet increased AT macrophage accumulation and AT inflammation.

**Figure 6:**
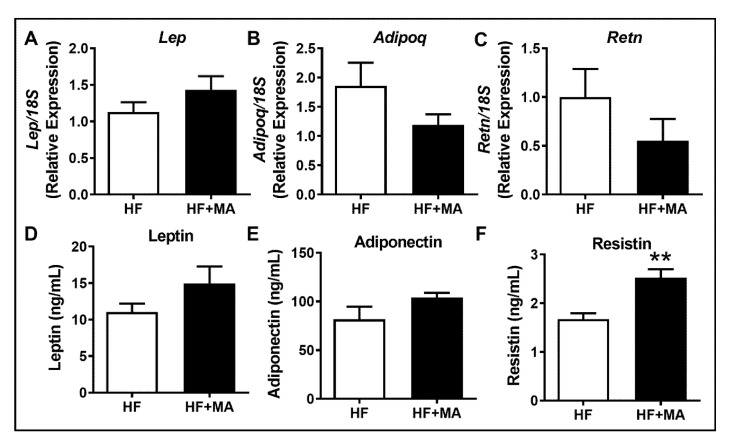
**Effect of dietary myristic acid on adipokines.** Real-time PCR analysis was performed in the VAT for the mRNA levels of *Lep*, *Adipoq*, and *Retn*, encoding leptin, adiponectin, and resistin, respectively (**A**–**C**). Plasma levels of these adipokines were determined by multiplex analysis (**D**–**F**). Values are expressed as mean ± SEM of 7–8 samples in each group. ** *p* < 0.01 vs. HF. HF, high fat; HF+MA, high fat + myristic acid.

*Effect of dietary myristic acid on adipokines*. In addition to AT inflammation, certain adipokines secreted by the AT also regulate systemic IR. Therefore, we next analyzed the mRNA levels of genes encoding for leptin, adiponectin, and resistin. We did not see a significant difference in these genes between HF and HF+MA-fed mice (Figure 6A–C). The analysis of plasma levels of these adipokines showed no difference in leptin and adiponectin levels (Figure 6D,E). Interestingly, we noted a significant increase in the circulating levels of resistin, an adipokine known to promote IR (Figure 6F).

**Figure 7:**
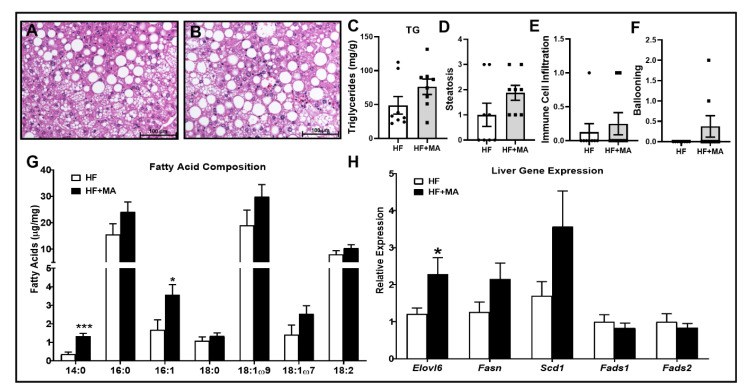
**Effect of dietary myristic acid on hepatic steatosis.** Liver sections from HF (**A**) and HF+MA (**B**), were stained with hematoxylin and eosin. Images were captured at 20× magnification using a Nikon Eclipse 80i microscope. Liver triglycerides (**C**) were measured by gas chromatography. Histology scores for the extent of steatosis (**D**), immune cell infiltration I(**E**), and ballooning (**F**) are shown. Fatty acid composition of liver TG is shown (**G**). Real-time PCR analysis was performed for the mRNA levels of genes involved in fatty acid synthesis, elongation, and desaturation (**H**). Values are expressed as mean ± SEM of 8 samples in each group. * *p* < 0.05 and *** *p* < 0.001 vs. HF. HF, high fat; HF+MA, high fat + myristic acid; 14:0, myristic acid; 16:0, palmitic acid; 16:1, palmitoleic acid; 18:0, stearic acid; 18:1ω9, oleic acid; 18:1ω7, vaccenic acid; 18:2, linoleic acid.

*Effect of dietary myristic acid on hepatic steatosis*. A HF diet not only promotes obesity but also leads to lipid accumulation in the liver. In order to determine whether MA alters the degree of steatosis, we first performed the hematoxylin and eosin (H&E) staining of liver sections. As shown in Figure 7A,B, the lipid accumulation trended towards an increase in HF+MA-fed mice compared to HF controls. Further quantification of liver TG by gas chromatographic analysis also showed a trend towards an increase in HF+MA-fed mice (Figure 7C). Although the chow diet-fed control is missing in this study, we previously showed that the TG levels in CD-fed mice were 7.1 ± 1.7 mg/g [31]. In the present study, the liver TG content in HF- and HF+MA-fed mice is 49 ± 13 and 76 ± 11 mg/g, respectively. Thus, there is a clear increase in liver TG upon HF diet feeding, which is enhanced in the presence of MA in the HF diet. We next analyzed the H&E stained sections for the extent of liver injury. Our histological analysis showed no difference in steatosis, immune cell infiltration, and ballooning (Figure 7D–F). Analysis of the fatty acid composition of liver TG showed a significant increase in MA levels in liver TG in HF+MA-fed mice compared to HF controls (Figure 7G), indicating that MA delivered via diet is indeed incorporated into TG. It has been reported that a HF diet itself increased MA in the liver compared to a control diet [11,32]. We previously showed that the MA level in chow diet-fed lean mice was very low (0.017 µg/mg tissue) [31]. On the other hand, our current study shows that the MA levels in the HF and HF+MA-fed mice are 0.37 and 1.33 µg/mg tissue, respectively (Figure 7G). These data suggest that the HF diet itself increases the MA level in liver TG, which is augmented by MA supplementation. Not only MA but also the incorporation of palmitoleic acid (16:1), which can be derived from the elongation and desaturation of MA, is increased in HF+MA-treated mice compared to controls. We next analyzed the liver mRNA for the expression of genes involved in fatty acid synthesis, elongation, and desaturation. Our data clearly show that the mRNA level of Eovl6 is significantly increased in HF+MA-fed mice compared to HF-fed controls (Figure 7H). However, no significant difference was noted in genes regulating fatty acid synthesis and desaturation.

**Figure 8:**
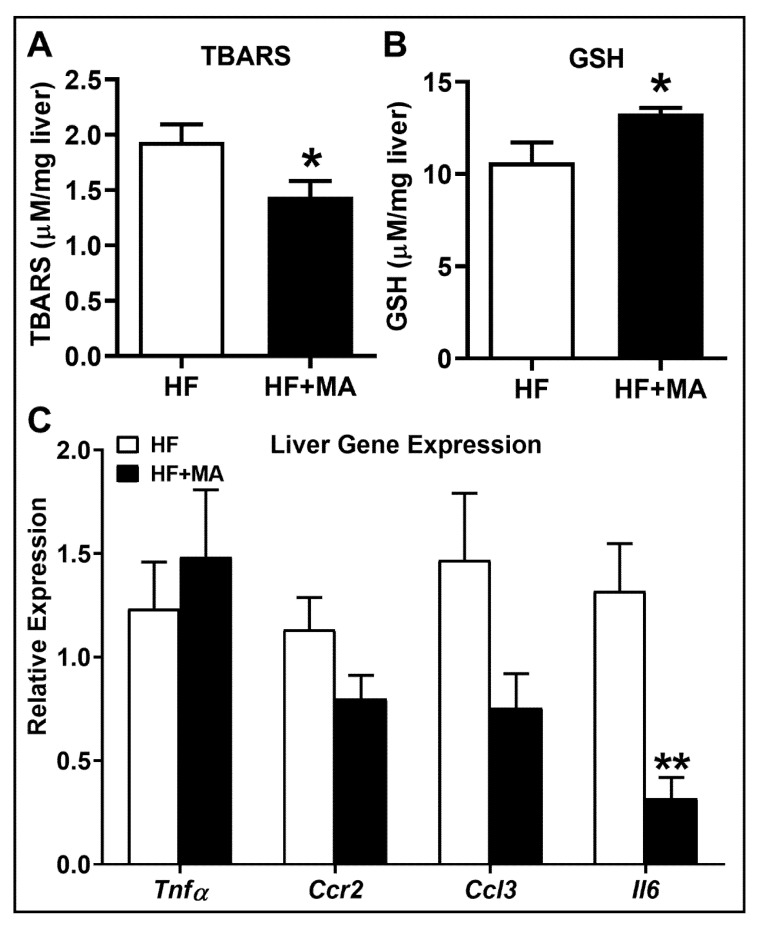
**Effect of dietary myristic acid on hepatic oxidative stress and inflammation.** The degree of lipid peroxidation was determined by measuring TBARS (**A**). Hepatic levels of total GSH were determined (**B**). Quantitative real-time PCR was performed in the liver for the mRNA levels of inflammatory genes (**C**). Values are expressed as mean ± SEM of 7–8 samples in each group. * *p* < 0.05 and ** *p* < 0.01 vs. HF. HF, high fat; HF+MA, high fat + myristic acid.

*Effect of dietary myristic acid on hepatic oxidative stress and inflammation*. We next assessed the oxidative stress markers in the liver. Analysis of TBARS levels showed a significant decrease in HF+MA-fed mice compared to HF controls (Figure 8A). Moreover, total glutathione, a measure of antioxidant status, showed a significant increase in HF+MA-fed mice (Figure 8B). These data suggest that MA reduced markers of oxidative stress in the liver. An analysis of liver inflammatory markers did not show a significant difference in the mRNA levels of *Tnfa*, *Ccr2*, and *Ccl3* (Figure 8C). Of note, the mRNA level of *Il6* showed a clear decrease in HF+MA-fed mice compared to controls (Figure 8C). Taken together, these data indicate that MA attenuates oxidative stress as well as inflammation in livers in obesity.

## 4. Discussion

Studies on the effect of different SFAs were conducted using an oil rich in a particular type of fatty acid. For example, palm oil and coconut oil have been widely used in various studies to determine the effects of long-chain and medium-chain saturated fatty acids on CVD and metabolic disorders. Very few studies were conducted using diets supplemented with purified fatty acids. Moreover, while a number of studies have addressed the role of palmitic acid in altering metabolic disorders, not much is known regarding the effect of MA, a rare fatty acid, in the body. Although MA is found in lower levels in the human body, it is gaining increased attention due to its association with obesity-related metabolic disorders and CVD. Milk fat or coconut oil is often used to enrich MA content in diet [33,34]. In one of these studies, MA content was altered in diets by supplementing with butterfat, hydrogenated coconut oil, and trimyristin, which provided about 1.1%, 1.7%, and 9.4% (wt/wt) MA, respectively. In another study, a diet enriched in MA providing 30% kCal was used [35]. We chose 3% (wt/wt) MA in the diet, which is within the range used in these studies. Using a HF diet supplemented with 3% myristic acid, we studied the specific effect of MA on altering obesity-linked metabolic disorders. We have demonstrated that a HF diet supplemented with MA increases body weight and systemic IR. MA promotes mild hypercholesterolemia, as evident from an increase in plasma total cholesterol levels. MA increases the VAT mass, VAT inflammation, as well as plasma resistin, an adipokine promoting IR. However, markers of oxidative stress and inflammation were improved in the livers of mice fed a HF+MA diet compared to HF controls. Taken together, our data suggest that MA supplementation leads to an impairment in obesity-linked IR but attenuates hepatic oxidative stress and inflammation.

One of the findings of this study is that a HF+MA diet increased body weight and VAT mass. It has been reported that serum MA is associated with weight gain in rats [36]. Several lines of clinical evidence also suggest that a link exists between MA and obesity. For example, MA intake is positively associated with body mass index, a measure of obesity, in human subjects [15]. Serum MA was higher in metabolically unhealthy individuals with obesity compared to lean, normal or metabolically healthy individuals [14]. However, the direct effect of MA on altering obesity is still unclear. Our data provide evidence that dietary MA increases body weight gain and VAT mass.

SFAs are known to promote IR and impair glucose metabolism. Palmitic acid is well-known for its effects in causing IR. However, not much is known regarding the role of MA on IR. Serum MA is associated with blood glucose levels in rats [36]. Ebbesson et al. showed that myristic acid levels in red blood cells are positively correlated to IR in human subjects [37]. Serum MA is positively correlated to the homeostatic model assessment for insulin resistance (HOMA-IR) index, a measure of IR, in Japanese patients with type 2 diabetes [38]. However, the causal role of MA in promoting IR is not clear from these studies. We provide evidence that a HF diet rich in MA promotes IR in mice. Regarding potential mechanisms, studies have linked IR to AT inflammation. Our data clearly show that F4/80, a macrophage marker as well as MCP1 and MIP1α, the inflammatory markers, were markedly increased in the HF+MA group compared to HF diet-fed mice (Figure 5A–F). A strong correlation exists between AT macrophage accumulation/AT inflammation and systemic IR [39,40]. Our findings indicate that AT macrophage accumulation and their polarization towards an M1 inflammatory phenotype, plays a role in mediating IR in HF+MA-fed mice. In addition, excess storage of SFAs in AT causes hypertrophy and hyperplasia of adipocytes. These adipocytes themselves exhibit impaired insulin signaling and also contribute to systemic IR by releasing SFAs [41]. It should also be pointed out that adipokines secreted by the adipocytes play an important role in altering IR. For example, while an increased circulating level of leptin is positively associated with IR, adiponectin is known to improve IR. Our data did not show a significant difference in these two adipokines. Interestingly, we noted a significant increase in plasma resistin levels in HF+MF-fed mice. Of note, resistin is another adipokine known to promote IR. For example, plasma resistin levels are positively correlated to obesity and IR in mice, and resistin neutralization using the leptin antibody improved IR in mice [42]. Moreover, the administration of antisense oligonucleotide against resistin attenuated the HF diet-induced IR in mice [43]. Overall, our data suggest that MA by increasing VAT mass, AT macrophage accumulation and inflammation, and resistin secretion from AT, contributes to the development of systemic IR.

Although MA is a rare fatty acid with respect to other SFAs, in particular, palmitic acid, it is gaining increased attention due to its hypercholesterolemic effects. Overall, SFAs favor the overproduction of LDL cholesterol [44]. The MA-rich diet raises plasma LDL cholesterol by stimulating LDL production without reducing LDL clearance in gerbils [22]. Moreover, human studies show that plasma MA is negatively associated with HDL cholesterol levels [45]. Of note, HDL is known for its role in promoting cardiovascular health. Further, Zock et al. have shown that a diet rich in MA increased LDL cholesterol and reduced the HDL to LDL ratio in human subjects [21]. An increase in both LDL and HDL cholesterol was also noted in human subjects [19]. Taken together, our study corroborates with these other studies and suggests that MA aberrantly regulates plasma cholesterol levels, which, in turn, can increase the CVD risk.

Obesity is strongly linked to NAFLD. NAFLD is characterized by an excess accumulation of triglycerides in the liver. SFAs, in particular, palmitic acid, are widely implicated in the development of NAFLD. For example, a single bolus of palm oil, rich in palmitic acid, increases hepatic triglycerides and increases the expression of NAFLD-related genes [46]. Moreover, Ogawa et al. have shown that palmitic acid-induced lipotoxicity is crucial for the development of NAFLD [47]. Regarding MA, serum MA is considered to be a predictor of non-alcoholic steatohepatitis. MA potentiates palmitic acid-induced lipotoxicity and steatohepatitis [35]. However, the direct effect of MA on altering the pathogenesis of NAFLD in obesity is still unclear. Our data showed a trend towards an increase in liver TG in HF+MA-fed mice compared to HF controls. However, our data showed that markers of hepatic oxidative stress and inflammation were attenuated in HF+MA-fed mice compared to HF controls. It should be noted, however, that HF+MA-fed mice showed an increase in palmitoleic acid (16:1) levels in liver triglycerides (Figure 7D). Of note, Guo et al. showed that this fatty acid promoted hepatic steatosis but reduced hepatic inflammation in mice [48]. Thus, an improvement in hepatic oxidative stress and a reduction in hepatic inflammation in the presence of hepatic steatosis can be attributed to an increase in palmitoleic acid in HF+MA-fed mice. Overall, while MA increases AT mass and AT inflammation, it exerts a differential effect on the liver.

One limitation of this study is the lack of the chow diet-fed groups. However, the choice of the isocaloric diet used in this study is helpful to assess the effect of MA in the context of obesity. Next, the role of endogenous MA in mediating the metabolic effects of the HF+MA diet cannot be ruled out. For example, a HF diet itself is shown to increase MA levels in the liver [11,32]. However, as mentioned, we fed mice a HF diet with similar diet composition. Therefore, it is reasonable to speculate that the de novo synthesis of MA may not alter much in livers or AT between HF and HF+MA groups and that the effect of MA seen in this study is primarily due to the diet.

MA is an important dietary fatty acid. For example, MA accounts for 18.9% of all SFAs in bovine milk. An increased dairy food intake by three serves/day for one month was associated with small increases in plasma levels of MA [49]. Further, increased uptake of butter is known to increase LDL cholesterol in humans [50]. MA is also rich in coconut oil and the consumption of coconut oil has been shown to increase LDL cholesterol in humans [51]. These various reports suggest that MA is an important component of dietary fat and increased consumption of a diet rich in MA is associated with features of metabolic syndrome.

## 5. Conclusions

In this study, we have demonstrated that dietary MA increases VAT mass, AT macrophage accumulation and inflammation in obesity. Further, we show that obesity-related IR is enhanced in mice receiving the MA-supplemented diet and it was associated with an increase in circulating resistin, an adipokine mediating IR. Our data also show that the MA diet increases plasma cholesterol levels to some extent. On the other hand, markers of hepatic oxidative stress and inflammation are attenuated in mice receiving the HF+MA diet. Taken together, our data suggest that MA promotes IR via increased VAT mass, M1 macrophage accumulation and inflammation in AT, and resistin secretion by AT. Our study provides important information about the specific effects of MA in obesity-related metabolic disorders. Our findings underscore the importance of the cautious use of MA-rich diets in patients with type 2 diabetes and/or CVD complications.

## Figures and Tables

**Table 1:** Diet Composition.

**Ingredients**	**High Fat**	**High Fat + MA**
	g	
**Protein**		
Casein	200	200
L-Cystine	3	3
**Carbohydrate**		
Corn Starch	72.8	72.8
Maltodextrin 10	100	100
Sucrose	172.8	172.8
Cellulose, BW200	50	50
**Fat**		
Soybean Oil	25	25
Lard	177.5	151.7555
Myristic Acid	0	25.7445
**Minerals and Vitamins**		
Mineral Mix S10026	10	10
DiCalcium Phosphate	13	13
Calcium Carbonate	5.5	5.5
Potassium Citrate, 1 H_2_O	16.5	16.5
Vitamin Mix V10001	10	10
Choline Bitartrate	2	2
**Total**	**858.15**	**858.15**

**Table 2:** Primers used in the study.

Gene (Abbr)	Description	Catalog Number
*18S*	18S ribosomal RNA	4352930E
*Adgre1 (EMR-1; F4/80)*	EGF-like module containing, mucin-like, hormone receptor-like 1	Mm00802529_m1
*Adipoq*	Adiponectin, C1Q, and collagen domain	Mm00456425_m1
*Ccl2*	Chemokine ligand 2/monocyte chemotactic protein 1	Mm00441242_m1
*Ccl3*	Chemokine ligand 3/macrophage inflammatory protein 1alpha	Mm00441258_m1
*Ccr2*	Chemokine receptor/monocyte chemotactic protein 1 receptor	Mm99999051_gH
*Chil3*	Chitinase-like 3	Mm00657889_mH
*Clec10a*	C-type lectin domain family 10, member A	Mm00546125_g1
*Elovl6*	ELOVL fatty acid elongase 6	Mm00851223_s1
*Fads1*	Fatty acid desaturase 1	Mm00507605_m1
*Fads2*	Fatty acid desaturase 2	Mm00517221_m1
*Fasn*	Fatty acid synthase	Mm01253292_m1
*Il6*	Interleukin 6	Mm00446190_m1
*Il10*	Interleukin 10	Mm99999062_m1
*Lep*	Leptin	Mm00434759_m1
*Mgl2*	Macrophage galactose N-acetyl-galactosamine–specific lectins 2	Mm00460844_m1
*Mmp3*	Matrix metallopeptidase 3	Mm00440295_m1
*Mmp12*	Matrix metallopeptidase 12	Mm00500554_m1
*Retn*	Resistin	Mm00445641_m1
*Saa3*	Serum amyloid A3	Mm00441203_m1
*Scd1*	Stearoyl-CoA desaturase 1	Mm00772290_m1
*Tnf*	Tumor necrosis factor, alpha	Mm00443258_m1

## Data Availability

The data presented in this study are available in the main text, figures, tables.
